# Emerging new roles of the lysosome and neuronal ceroid lipofuscinoses

**DOI:** 10.1186/s13024-018-0300-6

**Published:** 2019-01-16

**Authors:** Anil B. Mukherjee, Abhilash P. Appu, Tamal Sadhukhan, Sydney Casey, Avisek Mondal, Zhongjian Zhang, Maria B. Bagh

**Affiliations:** 1 Section on Developmental Genetics, Program on Endocrinology and Molecular Genetics, Eunice Kennedy Shriver National Institute of Child Health and Human Development, The National Institutes of Health, Bethesda, Maryland 20892-1830 USA; 2 Present address: Institute of Psychiatry and Neuroscience, Xinxiang Medical University, Xinxiang, 453003 Henan China

**Keywords:** Neurodegeneration, Neuronal Ceroid Lipofuscinosis, Batten Disease, Lysosomal Storage Disease

## Abstract

Neuronal Ceroid Lipofuscinoses (NCLs), commonly known as Batten disease, constitute a group of the most prevalent neurodegenerative lysosomal storage disorders (LSDs). Mutations in at least 13 different genes (called *CLN*s) cause various forms of NCLs. Clinically, the NCLs manifest early impairment of vision, progressive decline in cognitive and motor functions, seizures and a shortened lifespan. At the cellular level, all NCLs show intracellular accumulation of autofluorescent material (called ceroid) and progressive neuron loss. Despite intense studies the normal physiological functions of each of the *CLN* genes remain poorly understood. Consequently, the development of mechanism-based therapeutic strategies remains challenging. Endolysosomal dysfunction contributes to pathogenesis of virtually all LSDs. Studies within the past decade have drastically changed the notion that the lysosomes are merely the terminal degradative organelles. The emerging new roles of the lysosome include its central role in nutrient-dependent signal transduction regulating metabolism and cellular proliferation or quiescence. In this review, we first provide a brief overview of the endolysosomal and autophagic pathways, lysosomal acidification and endosome-lysosome and autophagosome-lysosome fusions. We emphasize the importance of these processes as their dysregulation leads to pathogenesis of many LSDs including the NCLs. We also describe what is currently known about each of the 13 *CLN* genes and their products and how understanding the emerging new roles of the lysosome may clarify the underlying pathogenic mechanisms of the NCLs. Finally, we discuss the current and emerging therapeutic strategies for various NCLs.

## Background

In 1955, Christian de Duve, while investigating the mechanism of insulin action, discovered the lysosome [1], a membrane-bound organelle which plays critical roles in the degradation and recycling of material delivered to it from both extracellular and intracellular sources [2–4]. Although historically known as the center for cellular waste disposal, the lysosome has since been reported to serve essential roles in such functions as cellular nutrient sensing, energy metabolism and plasma membrane repair. To maintain homeostasis, a network of lysosomal proteins, soluble lysosomal acid hydrolases, lysosome-related organelles, autophagosomes and other cellular constituents are put in place to degrade the material imported into the cell. Impairment of this lysosomal network contributes to a group of diseases called LSDs [5–7]. In most of the LSDs, neurodegeneration is a devastating manifestation [8]. Despite tremendous advances in the field, the precise molecular mechanism(s) underlying these diseases remain elusive. The emerging new roles of the lysosome as the nutrient sensor and signaling hub of the cell may provide a better understanding of the pathophysiology of various NCLs.

The lysosome, in addition to harboring the soluble acid hydrolases in its lumen, is endowed with numerous proteins that are localized to its membrane [9, 10]. For a long time, the lysosomal membrane was thought to provide just a barrier between the luminal acid hydrolases and the cytoplasm. However, emerging evidence indicates that the lysosomal membrane serves critical roles in a wide range of cellular functions, including phagocytosis, autophagy, membrane repair and apoptosis [10]. All lysosomal membrane proteins are synthesized in the endoplasmic reticulum (ER) and are selectively transported to the lysosome. The degradative functions of the lysosome are essential for cell clearance, signaling, metabolism and homeostasis. Cellular homeostasis also requires ubiquitylation, a process that facilitates specifying the proteins which are transported to the lysosomal lumen by endosomal sorting complex required for transport (ESCRT) to sort cargos tagged with ubiquitin into the invaginated endosomal membranes [11]. Recently, it has been reported that the ESCRT machinery plays an essential role in repairing injured endolysosomes and thereby providing a mechanism to protect the cells from death.

Compelling evidence indicates that the lysosome performs a much broader function than just being the cellular waste disposal. It has critical roles in some of the most vital processes like secretion, signaling, repair of the plasma membrane, and energy metabolism [12]. In addition, the lysosome has an essential role in autophagy [3, 4], which along with its other functions places this organelle at the center of several vital processes, such as nutrient sensing, metabolism and homeostasis [13]. Thus, impairment of lysosomal function has implications for the pathogenesis of all LSDs including the NCLs.

**Fig. 1:**
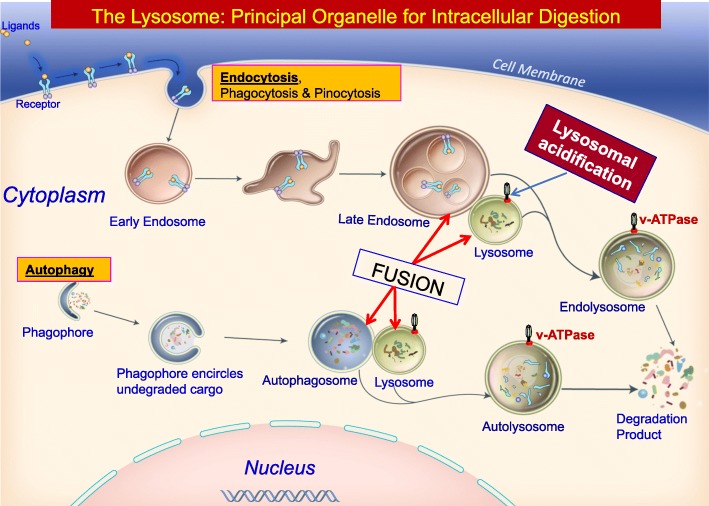
Dysregulation of the endocytic and autophagic pathways in LSDs including the NCLs. Cells import materials from outside the cell by endocytosis. This process is initiated by the invagination of the plasma membrane, which encloses the material forming the early endosome, which progresses to the late endosome. The fusion of the membranes of the late endosome and lysosome forms a hybrid structure called endolysosome. The fusion of the two membranes is catalyzed by Rab7, a small GTPase, and the endosomal cargo is degraded by lysosomal acid hydrolases and the constituent components are reutilized by the cell. The process of autophagy is initiated by the formation of a double-membrane structure in the cytoplasm called phagophore. This structure in its mature form is called phagosome, which encircles materials such as aged macromolecules and disabled organelles. The autophagosome fuses with the lysosome forming a hybrid structure called autolysosome. The lysosomal hydrolases then degrade the cargo to their respective components (i.e. amino acids for proteins, fatty acids for lipids, etc.) which are reutilized by the cells. Notably, impaired or missing lysosomal acid hydrolases or failure of endosome-lysosome and autophagosome-lysosome fusions may result in the accumulation of undigested cargo in the lysosome leading to lysosomal storage diseases

Lysosomes receive materials for degradation via three major pathways: phagocytosis, endocytosis and autophagy (Fig. 1). A network of lysosomal proteins, and other cellular constituents contribute to the degradation of the imported cargo delivered to this organelle [14]. In the following paragraphs, we articulate how the endolysosomal and autophagic pathways of degradation require lysosomal acidification and endosome-lysosome as well as autophagosome-lysosome fusions.

### The endolysosomal and autophagic pathways of degradation

The lysosome is the terminal organelle for degradation and cellular clearance. Materials from varying locations are delivered to the lysosome for degradation. There are two major pathways by which materials enter the lysosome. These are (i) endocytosis in which the material from outside the cell enters the lysosome via endocytic vesicles originating from the plasma membrane [15] and (ii) autophagy in which material from within the cell is transported to the lysosome via autophagosomes. It has been suggested that dysregulation of autophagy is a common mechanism in the pathogenesis of the LSDs including several NCLs and other more common neurodegenerative diseases [16].

The endo-lysosomal pathway plays multiple roles in cellular function and homeostasis [17, 18]. This pathway has an important function in enabling the cell to internalize extracellular macromolecules and fluids together with portions of the plasma membrane. It also plays vital roles in the sorting of the internalized membrane and its constituent proteins. The major sorting stations in this pathway consist of the early and late endosomes and pleiomorphic organelles with tubulo-cisternal and multi-vesicular domains, which are packed with intraluminal vesicles [19]. The cargo contained in the endosomes are either delivered to the lysosome where acid hydrolases catalyze degradation or promote recycling back to the plasma membrane via recycling endosomes, or further transported to the Golgi using the retromer complex [20, 21]. In some instances, the endosomes undergo fusion with the plasma membrane, thereby discharging the contents into the extracellular space. These extracellular materials are called the exosomes [22].

The Rab GTPases [23], which are the major regulators of vesicular trafficking, control the dynamics of sorting, trafficking and membrane remodeling in the endocytic pathway including secretion of the exosomes. Recent investigations have demonstrated how Rab35 and Arf6 might serve as input sensors for two types of endocytosis to balance membrane trafficking to preserve cell surface homeostasis. More recently, it has been shown that lysosomal contact with mitochondria regulates mitochondrial fission through the hydrolysis of Rab7, which allows bidirectional regulation of mitochondrial-lysosomal dynamics [24]. This may explain why lysosomal as well as mitochondrial dysfunction have been observed in various human diseases including the neurodegenerative LSDs like the NCLs. One of the important factors in intracellular digestion is lysosomal acidification, which directly impacts the catalytic activities of the lysosomal acid hydrolases as these enzymes work most efficiently in acidic pH.

### Lysosomal acidification

In virtually all NCLs, except *CLN2* and *CLN8*, lysosomal acidification is reported to be dysregulated [25, 26]. It has also long been recognized that acidification of the endocytic organelles is essential for the degradation and clearance of exogenous and endogenous material delivered to the lysosome via endosome and autophagosome pathways [15]. Lysosomal acidification is regulated by vacuolar ATPase (v-ATPase) [27–29], which is a multi-subunit protein complex consisting of a cytosolic V1-sector and a lysosomal membrane-anchored V0-sector. The v-ATPase acidifies a wide array of intracellular organelles by pumping protons across the membranes into the vesicular (late endosomal/lysosomal) lumen [27–29]. The V1-sector of v-ATPase generates energy by hydrolyzing ATP and this energy is utilized to facilitate the transport of protons from the cytoplasm across the lysosomal-membranes to the vesicular lumen. The recycling of the cell surface receptors, the receptor-ligand dissociation, protein degradation by lysosomal hydrolases, neurotransmitter loading as well as the recycling of synaptic vesicles are all pH-dependent [30, 31]. Moreover, it has been demonstrated that alterations in the intracellular pH alone severely alter organellar morphology, movement and function [32]. Indeed, it has been demonstrated that nanoparticle-mediated correction of lysosomal acidification defects may have implications for lysosomal-related diseases [33].

### The endosome-lysosome and autophagosome-lysosome fusion

An orderly execution of fusion of the membranous organelles is vitally important for the eukaryotic cells [34]. For example, the fusion of the autophagosome-lysosome and endosome-lysosome membranes is critical for the degradation of material imported from intracellular and extracellular sources that are delivered to the lysosome [4]. Similarly, membrane fusion plays an essential role in synaptic transmission in the central nervous system in which SNARE- and SM (Sec1/Munc18-like)-proteins have been reported to play critical roles [35]. The v-ATPase, by regulating lysosomal acidification, mediates the fusion of endocytic and autophagic vesicles although the mechanism(s) by which the fusion of these membranes occurs remains poorly understood [36]. Notably, the maturation of the autophagosome precursor has been shown to depend on homotypic membrane fusions [37].

Autophagy is a major degradative pathway in the cell [3, 4]. There are three different types of autophagy: (i) macroautophagy, (ii) microautophagy and (iii) chaperone-mediated autophagy. During macroautophagy (hereafter called autophagy), the cytoplasmic contents (e.g. long-lived proteins and disabled or aged organelles) are encircled by a double-membrane structure called the autophagosome [3, 4]. An autophagosome is a double-membrane structure, originating from the ER-mitochondria contact sites, called a phagophore, which encloses long-lived proteins, aged and disabled organelles, and aggregated proteins. Autophagosome fuses with the lysosomal membrane generating a hybrid organelle called autolysosome in which the acid hydrolases degrade the cargo. At the final stage of cargo-degradation, each type of autophagy requires the fusion of autophagosomes with functional lysosomes [38]. In recent comprehensive reviews [39, 40] the molecular definition of autophagy and autophagy-related processes as well as how their dysfunction may lead to neurodegeneration has been described. It has become increasingly evident that impaired autophagy is associated with several human neurodegenerative diseases including neurodegenerative LSDs like the NCLs [16, 40, 41]. Membrane fusion is an essential requirement for early biogenesis of autophagosome and degradation of cargo in the lysosome and this vital process is impaired in many neurodegenerative diseases [4, 40, 42].

Impaired membrane fusions have been reported in the LSDs as well as in common neurodegenerative diseases [43, 44]. Thus, fusion of these organelles is particularly important for maintaining neuronal survival as impaired autophagy causes accumulation of several aggregate-prone proteins (e.g. huntingtin, α-synuclein), which are potentially harmful to the neurons [44, 45]. Despite intense investigations the physiological functions of the mutant genes underlying various forms of the NCLs remain poorly understood. In the following paragraphs, we describe what is known about these genes, the proteins they encode and how the emerging new roles of the lysosome may advance our understanding as to how mutations in these genes may impair lysosomal function.

### *CLN* gene mutations and their differential pathologic manifestations in various NCLs

Commonly known as Batten disease [46–52], NCLs constitute a group of the most common inherited neurodegenerative LSDs that mostly affect children. Lysosomal accumulation of autofluorescent material (called ceroid), increased neuronal apoptosis, dysregulated autophagy, neurodegeneration and shortened lifespan are some of the common features shared by all NCLs. Our knowledge that the lysosome functions as a nutrient sensor and the signaling hub of the cell [12–14, 53–56] may be applied to facilitate a greater understanding of the pathogenic mechanism(s) underlying the NCLs.

The 13 different genes **(**Table 1), mutations of which cause various forms of NCLs, may be classified into four groups according to the proteins they encode. The group I genes (*CLN1, CLN2 CLN5, CLN10* and *CLN13*) encode lysosomal soluble proteins/enzymes. The group II genes (*CLN3, CLN7 and CLN12*) encode membrane proteins but two of the genes (*CLN6* and *CLN8*) encode ER-membrane proteins. The group III genes (*CLN4* and *CLN14*) encode soluble proteins and one gene in group IV (*CLN11*) encodes a protein in the secretory pathway [46–52]. A summary of the proteins encoded by all *CLN* genes is provided in Table 1. In Table 2, The *CLN* gene mutantions underlying pathophysiological manifestations of various NCL forms are provided in Table 2.

**Table 1:** Neuronal Ceroid Lipofuscinoses (Batten Disease)

Mutant Gene	NCL Disease	Encoded Protein	Classification and protein size	Posttranslational modification	Subcellular localization	Function	Interactions
*CLN1*	Infantile NCL (*CLN1*-disease)	Palmitoyl protein thioesterase 1 (PPT1)	soluble protein, 306 aa	N-gly M6P	Lysosomal lumen, extralysosomal vesicules, extracellular, presynaptic areas in neurons	Palmitoy-protein lthioesterase	S-acetylated proteins (GAP43, rhodopsin, saposin D)
*CLN2*	Late infantile NCL	Tripeptidyl peptidase 1 (TPP1)	soluble protein, 563 aa	N-gly M6P	Lysosomal lumen	Serine protease	*CLN3*, *CLN*5
*CLN3*	Juvenile NCL, Batten disease	*CLN3*/Batenin	6 TM protein, 438 aa	N-gly farnesylated phosphorylated	Late endosomal/lysosomal membrane, presynaptic vesicles	Unknown / predicted: pH regulation and modulation of vesicular trafficking and fusion	Hook1, kinesin-2, *CLN5*, Na+, K + ATPase, Rab7, fodrin
*CLN4*	Kuffs disease	Cysteine-string protein alpha (CSPα), DNAJC5	soluble protein, 198 aa	Palmitoylated	Cytosolic, vesicular membranes	Hsc70 co-chaperone involved in exocytosis and endocytosis	CSPα, SNAP-25, myosin IIB, calsenilin, DHHC17, dynamin-1, syntaxin, Gαs, Rab3b, synaptotagmin 9, Hsp70, Hsp40, Hsp90, HIP, HOP, SGT
*CLN5*	*CLN5* disease	–	soluble protein, 407 aa	N-gly M6P	Lysosomal lumen	Unknown / predicted: modulation of vesicular trafficking	PPT1/*CLN1*, TPP1/*CLN2*, *CLN3*, *CLN6*, *CLN8*
*CLN6*	*CLN6* disease	*CLN6*-protein	7 TM protein, 311 aa	None	ER-membrane (transmembrane)	Unknown	*CLN5*, *CLN6* CRMP-2
*CLN7*	Turkish variant of late-infantile NCL	MFSD8	12 TM protein, 518 aa	N-gly proteolytic cleaved	Lysosomal membrane	Predicted transmembrane transporter function predicted	AP-1, cathepsin L
*CLN8*	NCL 8	*CLN8*	5 TM protein, 286 aa	None	ER-membrane (transmembrane)	Unknown, predicted: to aid in the maturation of lysosomal proteins by transporting them from the ER to the Golgi complex, predicted regulation in lipid metabolism,	*CLN5*, *CLN8*, syntaxin 8, VAPA, GATE16, AGA, ARSA, ARSB, ARSG, CTBS, CTSA, CTSB, CTSD, CTSF, CTSZ, DNASE2, GALNS, GGH, GM2A, GNS, GUSB, HPSE, IDS, LIPA, MAN2B1, MAN2B2, MPO, NAGA, NEU1, PCYOX1, PLBD2, PPT1, PPT2, PSAP, RNASET2, SGSH, SIAW, SMPD1, TPP1
*CLN9*	–	Currently designated as *CLN4*	–	–	–	–	–
*CLN10*	Congenital NCL	Cathepsin D (CTSD)	soluble protein, 462 aa	N-gly M6P	Lysosomal lumen	Aspartyl protease	APP, CST3, CTSB, proSAP, and several others
*CLN11*	Unknown	Progranulin and granulins	soluble protein, 593 aa	None	Extracellular	Unknown/ predicted, roles in inflammation, embryogenesis, cell motility and tumorigenesis	MMPs, ADAMs, TGFα receptors, sortilin, ADAMTS-7/ADAMTS-12/perlecan/HDL/COMP, TGNFα receptors, EPHA2
*CLN12*	Unknown	ATPase 13A2, KRPPD, PARK9, HSA9947, RP-37C10.4	10 TM protein, 1180 aa	None	Lysosomal membrane	Unknown / predicted regulation of ion homeostasis	~ 43 vesicular trafficking and synuclein misfolding postulated proteins
*CLN13*	Unknown	Cathepsin F (CTSF)	soluble protein, 484 aa	N-gly M6P	Lysosomal lumen	Unknown / predicted: cysteine protease	CD47 antigen
*CLN14*	Unknown	Potassium channel tetramerization domain-containing protein 7 (KCTD7)	soluble protein, 289 aa	Phosphorylated	Cytosolic, partially associated to membranes	Unknown / predicted modulation of ion channel activity	Cullin-3, KCTD7

**Table 2:** Mutant *CLN* genes and underlying pathophysiology of various forms of NCLs

Mutant Gene	Cells/ tissues	Myoclonus & Seizures	Autofluorescent inclusions	Elevated lysosomal pH	ER Stress	Dysregulated degradation	Cellular dysfunction
*CLN1*	Ubiquitously expressed, CNS, brain	X	X	X	X	X	protein response
*CLN2*	Ubiquitously expressed, brain, neurons, cerebrospinal fluid	X	X			X	Endocytic pathway dysfunction
*CLN3*	Ubiquitously expressed, CNS, immune and circulatory systems, iPSC, neural progenitor cells, colorectal cancer cells	X	X	X	X	X	TGN is impaired, localized on the lysosome, cellular proliferation, apoptosis and synaptic transmission
*CLN4*	Ubiquitously expressed, neuronal synapses (1% of total synaptic vesicle-associated proteins)	X (Type A)	X	X	–	–	Type: B manifests movement abnormalities with dementia
*CLN5*	Ubiquitously expressed in human tissue, CNS, peripheral organs and tissues, neurons (ganglionic eminence) and microglia	X	X	X			Endosomal sorting, the stability of sortilin and CIMPR both declines, defective myelination
*CLN6*	Ubiquitously expressed	X	X		X	X	manifest characteristic cholesterol and subunit c of mitochondrial ATP synthase (SCMAS), aberrant biometal metabolism
*CLN7*	Ubiquitously expressed at a very low level, its expression in the liver, heart, and pancreas (markedly higher)	X	X	X		X	loss of *CLN7* causes depletion of soluble proteins in the lysosomes impairing reactivation of mTOR
*CLN8*	Ubiquitously expressed, high level expression in cerebral cortex and hippocampus in electrical kindling model of epilepsy	X	X		X	X	progressive motor neuron dysfunction and retinal degeneration, lysosomal β-glucosidase deficiency,
*CLN9*	Currently designated as CLN4	–	–	–	–	–	
*CLN10*	Ubiquitously expressed, brain	X	X	X		X	CTSD-processing
*CLN11*	Ubiquitously expressed, CNS, neuron, microglia, astrocytes, and endothelial cells	X	X	X	–	–	significantly activated microglia after TBI, the elevated lysosomal biogenesis in activated microglia, which increased cerebrocortical neuron damage, reduces lysosomal biogenesis
*CLN12*	Ubiquitously expressed, ventral midbrain, including substantia nigra (high lever), kidney and skeletal muscle (low level)	X	X	X	–	–	extrapyramidal involvement, oxidative-stress in neuroblastoma cells; dysregulated neurotransmission
*CLN13*	CTSF is expressed at a high level in cerebrocortical, hippocampal and cerebellar neurons	X	X	X	–	–	neurons showed accumulation of eosinophilic granules
*CLN14*	Ubiquitously expressed, cerebrocortical and cerebellar Purkinje cells, pyramidal cell layers of the hippocampus (high levels)	X	X	X	–	–	disrupt KCTD7-Cullin-3 interactions

### *CLN1*/PPT1

The discovery of an enzyme in bovine brain, its purification to homogeneity and the demonstration that it catalyzed the cleavage of the thioester linkage in palmitoyl-CoA and S-acylated H-Ras *in vitro* [57] paved the way for the identification of the mutant gene (now called *CLN1*) [58]. Inactivating mutations in the *CLN1* gene underlie infantile NCL (or INCL), also known as Santavuori-Haltia disease [59]. The *CLN1* gene encodes palmitoyl-protein thioesterases-1 (PPT1) [60], a soluble depalmitoylating enzyme, which is essential for the degradation of S-acylated proteins by lysosomal hydrolases [61]. Numerous proteins in the central nervous system undergo S-palmitoylation (or S-acylation), a process in which a 16-carbon fatty acid (predominantly palmitate) is attached to specific cysteine residues in polypeptides via thioester linkage [62]. It is the only reversible lipid modification that has emerged as an important regulatory mechanism for many proteins, especially in the brain [63, 64]. These S-acylated proteins require depalmitoylation by thioesterases prior to degradation by lysosomal acid hydrolases [61]. Thus, PPT1-deficiency impairs lysosomal degradative function causing intracellular accumulation of S-acylated proteins leading to INCL. At birth, the children afflicted with INCL are phenotypically normal. However, by 11-18 months of age they manifest signs of psychomotor retardation. By 2 years of age, these children are completely blind due to retinal degeneration. Around 4 years of age, an isoelectric electroencephalogram (EEG) attests to a vegetative state, which may last for several more years before eventual death [59].

**Fig. 2:**
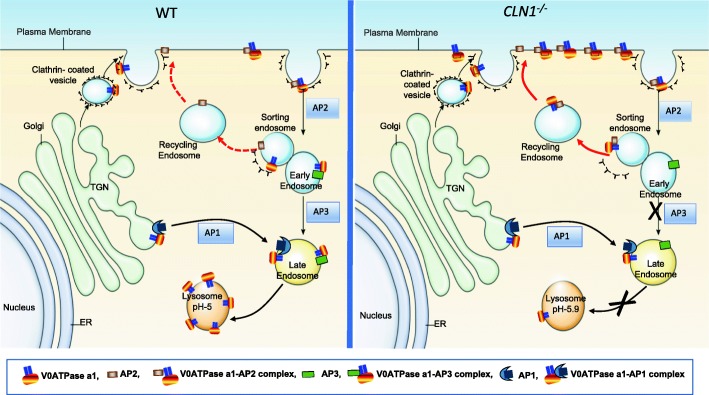
Dysregulation of lysosomal acidification in a mouse model of infantile NCL. Schematic representation of endosomal sorting and trafficking of a critical subunit of v-ATPase, the proton pump that maintains acidic pH of the lysosomal lumen. We recently uncovered that V0a1 requires S-palmitoylation for its endosomal transport to the lysosomal membrane (see ref. [67]). In *Cln1*^*-/-*^ mice (*right panel*), the lack Ppt1 causes misrouting of V0a1 to the plasma membrane instead of its normal location on the lysosomal membrane as seen in WT mice (*left panel*). Note that in *Cln1*^*-/-*^ mice, Ppt1-deficiency impairs the dissociation of V0a1 from AP-2, preventing its interaction with AP-3, which is essential for its transport from the sorting endosome to the late endosomal/lysosomal membrane. Consequently, the V0a1–AP-2 complex is misrouted to the plasma membrane via recycling endosome. This defect impairs v-ATPase activity, thereby dysregulating lysosomal acidification in neurons and other cells in *Cln1*^*-/-*^ mice. Since lysosomal hydrolases require acid pH for optimal catalytic activity, we propose that elevated lysosomal pH contributes to neuropathology in *Cln1*^*-/-*^ mice and most likely in INCL patients

It has been reported that *CLN1* mutations can also cause milder forms of INCL, which may manifest as late infantile, juvenile, or adult phenotypes [65, 66]. Although the precise biological roles of PPT1 and its *in vivo* substrates remain unidentified, a recent report suggested that cysteine string protein-α (CSPα) may be an *in vivo* substrate of PPT1 [67]. Notably, it has been demonstrated that *in vitro,* PPT1 depalmitoylates S-acylated growth associated protein 43 (GAP-43) and rhodopsin and its catalytic activity is higher at neutral pH (7.4) rather than at acidic pH (4.0) suggesting that PPT1 may have extra-lysosomal functions. Altered lysosomal pH has been reported in several NCLs including INCL [68]. It has recently been demonstrated that in *Cln1*^*-/-*^ mice, V0a1, a critical subunit of the v-ATPase (the proton pump of the cell) that regulates lysosomal acidification, requires S-palmitoylation for its lysosomal targeting [68]. Notably, in *Cln1*^*-/-*^ mice lacking Ppt1, V0a1 is misrouted to the plasma membrane instead of its normal localization on the lysosomal membrane (Fig. 2). This defect inhibits v-ATPase activity and consequently, alters the lysosomal pH in Ppt1-deficient cells [68].

Numerous studies have shown that both physiological and pathological conditions disrupt protein folding in the ER leading to the accumulation of these misfolded proteins, which cause ER-stress [69]. Increased ER stress activates a signaling network known as the unfolded protein response (UPR) to suppress protein synthesis [69]. In a wide variety of pathological conditions, including GM1 glycosidosis [70] and INCL [71], high levels of ER-stress have been reported to cause neuron loss. In response to ER stress, the protein-folding and degradation capacity of the ER is dynamically adjusted by the induction of the UPR [69]. Several comprehensive reviews have been published describing the prevalence of the UPR in the nervous system and its functional link in neurodegeneration [69, 71]. In *Cln1*^*-/-*^ mice, ER- and oxidative-stress have been shown to cause caspase activation leading to neuron loss by apoptosis [72–74]. Recently, a comprehensive review on ER-stress and UPR in various LSDs including the NCLs has been published [75].

### *CLN2*/TPP1

Mutations of the *CLN2* gene encoding tripeptidyl-peptidase 1 (TPP1) underlie pathogenesis of late infantile NCL (LINCL) or *CLN2*-disease [76]. TPP1 is a lysosomal protease that requires acidic pH for its activation. Moreover, inactivation of the aminopeptidase activity of TPP1 impairs the removal of tripeptides from the N-terminus of small proteins leading to *CLN2*-disease [77, 78]. Children with LINCL are phenotypically normal at birth but around 2-4 years of age the disease manifestation occurs, and they succumb to the disease when they are around 6-15 years old.

Like cathepsin D (CTSD), a lysosomal aspartyl protease, TPP1 is synthesized as an inactive proenzyme (pro-TPP1) in the ER. It is then autocatalytically processed to the active enzyme in the acidic pH of the lysosome requiring Ca^++^ [79, 80]. The *CLN2* gene is ubiquitously expressed and developmentally regulated [81]. In the human brain, TPP1 is expressed at a high level starting at 2 years of age [81] and in mice and rats, the highest expression level is reached at adulthood [82]. Like most of the NCLs, the management of the patients with *CLN2*-disease is palliative. It requires a multidisciplinary approach as the disease has a complex array of symptoms and its progression is generally rapid. Moreover, because of the rapid decline of the medical condition of these patients, it is essential that the patient families are given extensive psychosocial support. An excellent review article has recently been published in which the authors provided a detailed overview of *CLN2* disease and the associated complications that might be expected [83].

In both *CLN2*- and *CLN3*-diseases, endosomal/lysosomal trafficking as well as autophagy are reported to be dysregulated [84]. These abnormalities in *CLN2*-deficient cells may stem from oxidative-stress, which upregulates the PI3/Akt pathway activating the mechanistic (mammalian) target of rapamycin complex 1 (mTORC1) [85]. Using induced pluripotent stem cells (iPSCs) from normal subjects and *CLN2* patients, it has been shown that the earliest *CLN2* disease events can be recapitulated in the human iPSCs at the pluripotent stage and during neuronal differentiation [86]. Moreover, these authors have reported distinct, yet overlapping, early-stage pathology in multiple subcellular compartments. Furthermore, the results of this study provided the proof-of-principle that establishes a platform for the development of genotype-directed therapeutics for NCL by drug screening in patient neurons differentiated from iPSCs. Remarkably, when the iPSCs were differentiated to neurons, other abnormalities characteristic of the *CLN2*-disease began to appear [86]. Notably, while LSD features were not detectable in iPSCs, disease-specific storage materials were seen upon neural differentiation of the same cells. Recently, an unbiased exhaustive proteomic analysis of the brain and cerebrospinal fluid from patients with *CLN1*-, *CLN2*- and *CLN3*-disease have revealed significant alterations in the expression of proteins in each of these NCLs [87]. Studies like these are likely to identify biomarkers for these diseases, which are essential for evaluating emerging therapeutic strategies and to determine their efficacy.

### *CLN3*/ Batten disease

The *CLN3* gene was identified in 1995 and it was reported that mutations in this gene underlie juvenile NCL (JNCL) [88]. It encodes a 438-amino acid transmembrane lysosomal protein with both its N- and C-termini localized to the cytoplasm [89, 90]. The *CLN3* mutations as well as clinicopathological spectrums have previously been reviewed [91].

The most frequent disease-causing mutations in this gene, found in patients with *CLN3*-disease, is a 1 kb deletion that causes removal of exons 7 and 8, which generates a premature stop codon [88]. This mutation results in a substantial decrease in mRNA expression and stability. Therefore, it is likely that the mutant gene expressing a truncated *CLN3*-protein is not expressed at all [92]. In both humans and mice, the *CLN3*/*Cln3-*gene and *CLN3*/*Cln3*-protein are ubiquitously expressed [93]. It has been reported that mutations in the *CLN3* gene impair loss of vision, cause epileptic seizures, as well as progressive decline in motor and cognitive functions. However, there are reports providing evidence that *CLN3*-pathology also extends beyond the central nervous system for example, the immune- and circulatory systems [94–96]. Although lysosomal localization of *CLN3* has also been reported, it is also localized to the neuronal synapses and growth cones of telencephalic neurons of mice. Furthermore, *CLN3* has been shown to colocalize with the SV proteins such as SV2 and synaptophysin [97–99]. Thus, it has been suggested that *CLN3* may have a role in endosomal/lysosomal system as well as in neuronal synapses.

While the precise function of *CLN3* is not yet clearly understood, its localization primarily on late endosome and lysosome is suggestive of its important roles in these organelles [99, 100]. Notably, in *CLN3-*ablated HeLa cells expressing CD8-tagged CI-M6PR as the reporter, it has been demonstrated that in these cells the exit of CD8-CI-MPR from the TGN is impaired [101]. Recent reports indicate that *CLN3* plays a critical role in autophagy and this process is defective in *CLN3*-disease models [102]. Moreover, using juvenile *CLN3* disease patient-derived iPSCs and neural progenitor cells derived from juvenile *CLN3* disease it has been shown that autophagy is defective [103]. In addition to its localization on the lysosomal membrane and its role in the function of this organelle, *CLN3*-silencing has been shown to stimulate proliferation in human colorectal cancer cells [104]. Interestingly, it is reported that the wild, but not the mutant *CLN3*, binds galactosylceramide suggesting that it may transport galactosylceramide [105]. Moreover, it has also been demonstrated that suppression of galactosylceramide synthase by siRNA in normal cells adversely affected cellular growth and induced apoptosis. Notably, storage of subunit c of ATP synthase has also been reported in all NCLs except in INCL. However, it has been reported that the accumulation of subunit c does not cause the neuropathology characteristic of NCL-diseases [106]. Considering all these observations, it appears that *CLN3* mutation adversely affects several cellular processes such as lysosomal pH, endocytosis, autophagy, transport of proteins from the TGN, cell proliferation, apoptosis and synaptic transmission, although it remains unclear what precise biological function(s) *CLN3* regulates and what is the mechanism of such regulation. Recently, using human iPSC models of NCLs Lojewski and colleagues [86] have reported endocytic pathway dysfunction in cells carrying mutations in the *CLN2*- and *CLN3*-genes, respectively. Although phosphorylation of TFEB, which prevents its translocation to the nucleus, is catalyzed by mTORC1, it has recently been reported that in a *CLN3* disease model, TFEB activation has been achieved by the inhibition of Akt by trehalose, which mediated cellular clearance and neuroprotection [107].

### *CLN4* / DNAJC5

Mutations in the *CLN4* / *DNAJC5* gene encoding CSPα underlie the adult onset form of NCL also called Parry disease [108], is a very rare and difficult to diagnose NCL [106]. At least two variants of Kufs disease have been reported [109]. The type A variant is a progressive myoclonus epilepsy with cognitive impairment, and type B variant manifests movement abnormalities with dementia [110–112]. Although most of the NCLs are autosomal recessive diseases, the inheritance pattern of type A Kufs disease (*CLN6* mutations) is autosomal dominant [112]. Proper folding of proteins plays critical roles in their function and it has been reported that CSPα acts as a chaperone to facilitate correct folding of proteins [113]. Thus, its localization in the neuronal synapses accounts for 1% of total synaptic vesicle-associated proteins. In the neuronal synapses, α-synuclein (α-Syn) and CSPα are present in abundance [114]. Although it has been reported that dominant mutations in *α-SYN* gene cause Parkinson's disease, the physiological role(s) of α-Syn remains elusive. Targeted-disruption of the *CSPα* gene results in progressive neurodegeneration in mice and it has been suggested the co-chaperone function of CSPα is required for the survival of neurons [115]. Remarkably, it has been found that transgenic expression of α-Syn prevents neurodegeneration caused by CSPα-ablation. These results demonstrate not only the neuroprotective role of α-Syn, but also how CSPα works in concert with α-Syn in the nerve terminals to protect the neurons [115]. Interestingly, a recent study has reported that DNAJC5/CSPα and PPT1/*CLN1*, which is mutated INCL, may be functionally linked [67].

### *CLN5*

The *CLN5* gene encodes a soluble lysosomal glycoprotein of unknown function. Although its mutations were first discovered in the Finish variant of late infantile NCL, they are now reported in both juvenile and adult NCL patients of a wide range of ethnicities [116, 117]. *CLN5*’s expression varies throughout the body, having the highest levels in the central nervous system and moderate levels in the peripheral organs and tissues. In the brain, it is highly expressed in the cerebral cortex and cerebellum [118]. Similar expression patterns occur in the mouse brain where it is also developmentally regulated. Moreover, it is expressed in the ganglionic eminence and microglia, the latter of which sees very early activation in *Cln5*^*-/-*^ mice, which may adversely affect adjoining normal neurons. In addition, *CLN5*-protein has been reported to localize to the lysosomal compartment [119] and *CLN5* overexpression in COS-1 cells has been shown to interact with *CLN1*/PPT1, *CLN3, CLN6, CLN8*, and endogenous *CLN2*/TPP1 [120].

Although the precise function of *CLN5* encoded protein remains unclear, it has been reported that it plays a role in endosomal sorting [121]. Indeed, it has been shown that in HeLa cells, overexpressing HA-tagged *CLN5* and myc-tagged sortilin, a lysosomal enzyme transporter, both are coimmunoprecipitated. This suggested an interaction between these two proteins. Conversely, in *CLN5*-deficient cells the stability of sortilin and CIMPR both declined [122]. The importance of *CLN5* in the brain is suggested by its relatively high expression in neurons and microglia [118]. Moreover, patients with late infantile *CLN5*-disease and in the brains of *Cln5*^*-/-*^ mice, defective myelination occurs in the brain [118]. Further investigations are likely to delineate the precise physiological function(s) of the *CLN5* gene product.

### *CLN6*

Mutations in the *CLN6* gene cause classical and variant late infantile NCL [123, 124]. *CLN6* encodes a 27 kDa transmembrane ER-protein of 311 amino acids, which is expressed in virtually all tissues, including the cerebellum and the hypothalamus. However, its biological function(s) remain obscure and there is currently no known protein homologue of *CLN6* [125]. The expression of *Cln6* is developmentally regulated in the murine brain and mice aged P14 and older express *Cln6-*mRNA in cells of the cerebral cortex (layers II-VI), cerebellar Purkinje cells and in the dentate gyrus of the hippocampus. Moreover, it has been reported that *CLN6* has a role in the regulation of cellular acidification, endocytosis and autophagy [126–128]. *CLN6* mutations also manifest characteristic NCL phenotypes in lysosomal storage granules such as intracellular autofluorescent lipopigments (constituent of ceroid), cholesterol and subunit c of mitochondrial ATP synthase (SCMAS) [106, 126]. However, SCMAS are absent in lipopigments of *CLN1*, *CLN4* and *CLN10*. It remains unclear if SCMAS are present in the lipopigments in *CLN11-CLN14*. Notably, it has been reported that there is no difference in the total mannose 6-phosphate proteome in brain tissues from patients with *CLN6*-disease and healthy controls [129]. One of the striking findings in *CLN6*-disease is the aberrant biometal metabolism, which is also reported in common neurodegenerative disorders such as Parkinson’s and Alzheimer’s [130]. Biometals such as copper, zinc, manganese and cobalt have been shown to accumulate in animal models of *CLN6* disease [130]. In *Cln6* mutant (*nclf*) mice, accumulation of these biometals has been reported in the cerebral cortex, spinal cord, liver and heart [131]. Further investigations are required to determine the biological function(s) of *CLN6* and how impaired function of this gene leads to the accumulation of biometals and how this defect leads to *CLN6* disease pathogenesis.

### *CLN7*

Mutations in the *MFSD8* gene cause *CLN7*-disease, which is a variant late infantile phenotype [132]. Although *MFSD8* is ubiquitously expressed at a very low level, its expression in the liver, heart and pancreas is markedly higher. Interestingly, rat neurons, astrocytes and microglia as well as cultured microglial cells showed much higher levels of *Mfsd*8-mRNA [133]. The abundance of *Mfsd*8-mRNA was more pronounced in the cerebral cortex and the midbrain and much less pronounced in the hippocampus [133]. MFSD8 is a multispan integral lysosomal membrane protein belonging to the major facilitator superfamily (MFS) of active permeases. These proteins function as transporters of sugars, sugar phosphates, drugs, inorganic and organic cations, amino acids and neurotransmitters across membranes. However, its precise biological function remains unclear. Results from colocalization studies of MFSD8 and lysosomal membrane markers suggest lysosomal localization of this protein [134]. Studies using lysotracker red and Lamp-1 colocalization in HeLa cells with GFP-tagged MFSD8 found similar results [135]. Remarkably, none of the pathogenic MFSD8 mutations had any adverse effect on protein trafficking or lysosomal localization [133]. It has been reported that in *Cln7*-KO mice, loss of *Cln7* in the brain leads to lysosomal dysfunction and impairs constitutive autophagy leading to neurodegeneration late in the disease process [136]. It has also been demonstrated that *CLN7*-protein is cleaved by lysosomal proteinases generating N- and C-terminal fragments, which are then released in the extracellular space. Loss of *CLN7* causes depletion of soluble proteins in lysosomes impairing reactivation of mTOR [137], which is a potent anabolic regulator of cell growth and metabolism. Further investigations are essential to delineate the specific role(s) of *CLN7* in neurons and how MFSD8 mutations may lead to *CLN7* disease.

### *CLN8*

In humans, *CLN8* mutations manifest two distinct phenotypes. One of these phenotypes is characterized by progressive seizures and mental retardation and the other is a juvenile-onset variant, which is called Northern Epilepsy [138]. The *CLN8* gene encodes an ER membrane-spanning protein containing several hydrophobic domains. The protein is reported to shuttle between the ER and Golgi intermediate compartment for recycling. In non-neuronal cells, *CLN8* contains an ER-retrieval signal KKRP (Lys-Lys-Arg-Pro) at the carboxy terminal [139, 140]. The naturally occurring mouse model of the disease called *mnd* (motor neuron degeneration) shows progressive motor neuron dysfunction and retinal degeneration [140]. *CLN8*-mRNA is expressed ubiquitously with high level expression in cerebral cortex and hippocampus in electrical kindling model of epilepsy [141]. However, the relevance of *CLN8* overexpression in this model still needs to be determined. Although the precise function of *CLN8* remains unclear, a 200-amino acid (residues 62–262) TLC (TRAM-LAG1-*CLN8*) domain has been suggested to be essential for ceramide synthesis, glycosphingolipid trafficking [140] and lipid homeostasis [142, 143]. Of note, a report suggested *CLN8* as a genetic modifier after the stimulation of *CLN8*-mRNA expression in a chemically induced Gaucher disease model [144]. However, the mechanism by which *CLN8* expression is induced in this model, which is caused by lysosomal β-glucosidase deficiency, remains unclear. Interestingly, several studies suggested that altered *CLN8* function may be linked to ER- and oxidative-stresses (reviewed in [75]), disruption of calcium homeostasis, defective mitochondrial function, inflammation and apoptosis [145–147]. A recent study demonstrates that *CLN8* protein is retrieved from the Golgi complex to the ER via coat-protein I (COPI) and that mutations in the *CLN8* gene impairs the transport of lysosomal enzymes such as TPP1 leading to pathogenesis of *CLN8*-disease [148]. Undoubtedly, further research is needed to understand the mechanism(s) of pathogenesis of *CLN8*-disease.

### ***CLN9*/**reclassified as *CLN4*

#### *CLN10*/ CTSD

Mutations in the *CLN10* gene cause a severe neurodegenerative LSD called congenital NCL (CNCL) [149, 150]. Clinically, congenital NCL (*CLN10* disease) manifests with respiratory insufficiency after birth and status epilepticus, which are followed by death within hours to weeks ([149] and references there in). The *CLN10* gene encodes cathepsin D (CTSD; EC 3.4.23.5), an aspartic protease belonging to the pepsin superfamily. It is associated with several physiological processes such as protein degradation, autophagy and apoptosis [151]. CTSD hydrolyzes a wide variety of substrates including the extracellular matrix proteins fibronectin and laminin. However, the *in vivo* substrates of this enzyme have not been clearly identified although it has been reported that CTSD catalyzes the cleavage of α-synuclein [152], a protein associated with Parkinson’s disease.

Human subjects with *CLN10*-disease either die prenatally or within a few weeks after birth. Typically, these patients present with microcephaly and seizures and unlike other forms of NCLs they lack the progressive cognitive/motor or visual deficits [153, 154]. Sporadic mutations in the *CLN10* gene causing CTSD deficiency in sheep have been reported as congenital ovine NCL [155] and in American bulldogs [156].

Mammalian CTSD is synthesized as a 53 kDa pre-pro enzyme protein [157]. The pre-pro-CTSD is first proteolytically processed to a 48 kDa proenzyme, which then is transported through the endosomal pathway to the lysosome. Here the proenzyme is further processed by the proteolytic actions of cathepsin B and L, generating a 31 kDa and a 14 kDa fragment; non-covalent dimerization of these two fragments constitutes the mature, catalytically active-CTSD enzyme [157]. Variable pH optima have been reported for CTSD but maximal enzymatic activity is manifested at a pH optimum of 3.5 (26). Purified CTSD from porcine spleen has shown a pH optimum near 3 and 4, which has been reported to vary with salt concentration [158].

Several animal models with inactivating mutations in the *CLN10/CTSD* gene manifest CNCL phenotype. Targeted-disruption of the *Cln10*/*Ctsd* gene has been reported to cause an early-onset NCL phenotype and progressive neurodegeneration in mice and *Drosophila* [159–162]. It has also been reported that CTSD-processing is defective in lysosomes derived from the brain of *Cln1*^*-/-*^ mice, which suggests that lysosomal deficiency of enzymatically active CTSD is a common pathogenic link between INCL and CNCL [163].

#### *CLN11*/PGRN

The *CLN11* gene encodes progranulin (PGRN). In 2012, mutations in the PGRN gene were first reported in two siblings suffering from an adult-onset NCL [164]. PRGN was originally described as a growth factor that regulates wound healing, vasculogenesis and tumor growth [165]. However, in 2006, landmark studies showed that mutations in the *GRN* gene also underlie a familial form of frontotemporal lobar degeneration (FTLD) with distinct neuropathological features consisting of ubiquitin-positive protein aggregates in the nucleus and cytoplasm of cortical neurons [166]. Subsequently, these aggregates were found to be enriched in TAR DNA-binding protein-43 (TDP43) [167]. Moreover, it was discovered that patients with homozygous GRN mutations developed NCL-11 [164, 168]. Interestingly, while heterozygous mutations in the granulin gene (*GRN*) in older adults lead to haploinsufficiency in PGRN, which causes FTLD, homozygous mutations in the *GRN* gene lead to complete PGRN loss, which causes *CLN11*-disease in children [169]. However, the cause of the differential pathologic manifestation remains unclear.

Within the central nervous system *GRN*-mRNA is expressed in a variety of cell types including neuron, microglia, astrocytes and endothelial cells [170]. Despite its potential role in lysosomal function, the presence of a secretory signal in the N-terminal of GRN protein facilitates its regulated secretion [171]. Interestingly, microglial activation enhances progranulin expression and significantly impacts neuronal function and synaptic density [172]. Extracellular progranulin can bind to multiple receptors including sortilin, which transports it to the lysosome [173]. Progranulin also binds to TNFα-receptor [174] as well as the ephrin type-A receptor (EPHA2) on the cell surface and such interactions activate tyrosine kinase activity of EPHA2 and downstream kinase Akt [175]. This may suggest that signaling pathways downstream of Akt may also be activated by progranulin.

Although PGRN has been shown to be transported to the lysosome via sortilin [176], its function(s) in this organelle remains unclear. In macrophages, granulins, cleaved from PGRN, bind to CpG oligodeoynucleotides in lysosomes, enabling Toll-like receptor-9 signaling [177]. Recently, it has been reported that in the mouse traumatic brain injury model, PGRN prevents lysosomal dysfunction [178]. Most notably, the expression of PGRN is significantly elevated in activated microglia after traumatic brain injury. The elevated lysosomal biogenesis in activated microglia, which increased cerebrocortical neuron damage, reduces lysosomal biogenesis [54, 179]. It has been reported that PGRN-deficiency causing lysosomal dysfunction can be explained based on lipidomic and transcriptomic considerations [180]. In a recent comprehensive review, Paushter and colleagues have provided new insights into the lysosomal function of PGRN and its link to multiple neurodegenerative diseases [181]. More research would be needed to advance our understanding of the role of PGRN in the pathogenesis of *CLN11*- and FTD-diseases.

#### *CLN12*/ATP13A2

The *CLN12* disease is caused by loss of function mutations in the predominantly neuronal P-type ATPase (*ATP13A2*) gene. The *CLN12* (*ATP13A2*) gene is also known as *KRPPD*, *PARK9*, *HSA9947*, *RP-37C10.4*. It encodes a 36 kDa lysosomal transmembrane protein containing 10 predicted transmembrane domains [182], which were previously shown to underlie a rare form of autosomal recessive juvenile-onset Parkinson dementia called Kufor-Rakeb syndrome [183]. The patients afflicted with this syndrome manifest characteristics of not only typical NCL, but also show extrapyramidal involvement. Postmortem pathological examination of the brain tissues from a Kufor-Rakeb syndrome patient with homozygous missense mutations in the *ATP13A2* gene showed extensive deposition of lipofuscin in the retina, cerebral cortex, basal ganglia and cerebellum [183]. In most human tissues, *CLN12*-mRNA is detectable, but it is expressed at a high level in the ventral midbrain, including substantia nigra, and to a lesser extent in the kidney and skeletal muscle [182].

*CLN12/ATP13A2* gene product is targeted to the acidic compartments of the cell including the late endosome and lysosome [182, 183]. Cultured fibroblasts derived from patients with Kufor-Rakeb syndrome as well as ATP13A2-deficient cell lines have demonstrated that loss of this protein impairs lysosomal acidification, which in turn impairs the degradation of cargo by lysosomal hydrolases disrupting lysosome-mediated clearance of the autophagosomes [182, 184]. Recently, it has been uncovered that impaired *CLN12*/ATP13A2 function causes oxidative-stress in human neuroblastoma cells [185]. Interestingly, oxidative-stress is found to increase the expression of the *CLN12/ATP13A2-*mRNA [186]. A comprehensive review on this subject has been published linking its role as a cation transporter regulating Mn^2 +^, Zn^2 +^, Mg^2 +^ homeostasis with H^+^ ions concentration in the cell [187]. A pathogenic link has been suggested between *CLN12*/ATP13A2 and Parkinsonism as they both protect neurons against α-Syn toxicity. Since cation regulation and homeostasis are vital for neuronal function including intra- and inter-cellular signaling [188], the loss of transporter function of the ATP13A2 may explain the dysregulated neurotransmission and eventual dementia characteristic of *CLN12* disease.

#### *CLN13*/CTSF

The *CLN13* gene encodes cathepsin F (CTSF) and mutations in this gene were originally reported in mice [189], which develop neurological disease with accumulation of autofluorescent material in neurons of the cerebral cortex, hypothalamus, cerebellar Purkinje cells and other regions of the brain. The neurological disease develops between 12–16 months of age and is characterized by the lack of coordination, muscular weakness and premature death. The pathological findings in the brain also include numerous activated microglial cells [189]. Recently, 3 families with adult-onset NCL causing dementia and motor disturbances without epilepsy have been described [190, 191]. These patients carried rare mutations in the *CTSF* gene, which were identified after linkage analyses, and exome sequencing. CTSF is a cysteine protease consisting of 484 amino acids. It contains a 251-amino acid pro-peptide consisting of a N-terminal cystatin-like pro-region, which works as a cysteine protease inhibitor [192]. Synthesized in the ER, CTSF is tagged with mannose 6-phosphate residues in the cis-Golgi and transported by CI-M6PR to the late endosomal/lysosomal compartment [193]. CTSF is expressed at a high level in cerebrocortical, hippocampal and cerebellar neurons [190]. Although CTSF is recognized as a lysosomal cysteine proteinase, its *in vivo* function remains obscure. Targeted-disruption of the *CTSF* gene in mice has been carried out and phenotypic characterization of the *CTSF*^*-/-*^ animals shows that they are apparently healthy and reproduced normally. However, these mice manifested progressive weakness in their hind legs and declining motor coordination at 12 to 16 months of age. This was followed by significant weight loss leading to death. Pathologic analysis of the CTSF-deficient neurons showed accumulation of eosinophilic granules that had characteristics of lysosomal lipofuscin as well as elevated levels of autofluorescent lipofuscin, which are characteristic of all NCLs. These findings may indicate that CTSF either only mildly affects the phenotype or is mildly compensated by other gene product(s). Thus, the phenotypic manifestation of CTSF-deficiency requires a longer period for the manifestation of *CLN13*-disease symptoms [190].

### *CLN14*

Progressive myoclonic epilepsy (PME) is a clinically defined epileptic syndrome that manifests as myoclonic seizures and progressive neurological dysfunction [194, 195]. Mutations in the potassium channel tetramerization domain-containing protein 7 (KCTD7) have been extensively linked to progressive myoclonic epilepsy [196]. Moreover, homozygous mutations in the KCTD7 gene have been reported to cause a subtype of NCL discovered in two siblings in a Mexican family who presented with infantile-onset, progressive myoclonic epilepsy, cognitive impairment, loss of vision, motor regression, and premature death. Pathological analysis showed prominent NCL-type storage material [197]. Further analyses showed that these patients carried a missense mutation in the *KCTD7* gene and pathological analysis showed autofluorescent storage material characteristic of the NCLs. Currently, *KCTD7* gene represents *CLN14* [197].

KCTD7 is a member of the KCTD protein family [198]. It is a highly conserved protein consisting of 289 amino acids, and in the mouse, it is predominantly expressed in the cerebrocortical and cerebellar Purkinje cells as well as in the pyramidal cell layers of the hippocampus [197]. It is a soluble cytosolic protein. The structure and localization of KCTD7 in various organs suggests that it is involved in hyperpolarization of the cell membrane via interaction with a component of the ubiquitin ligase complex [198]. In a patch clamp study, it has been demonstrated that KCTD7 overexpression in murine neurons hyperpolarizes the cell membrane and decreases excitability of these cells. Moreover, KCTD7 directly interacts with Cullin-3, a component of E3 ubiquitin-protein ligases, for degradation by ubiquitin-proteasome system. Furthermore, missense mutations in the KCTD gene found in *CLN14* disease, disrupt KCTD7-Cullin-3 interactions, which suggests that such mutations may impair cellular degradative process [197, 198].

### Emerging new roles of the lysosome in the context of NCLs

How might emerging new roles of the lysosome clarify the differential pathologic manifestations in the NCLs ? The lysosome has been known as the major degradative and recycling organelle in the cell. However, it is increasingly being recognized as a “command and control center for cellular metabolism” [199]. A clear understanding of the emerging new roles of the lysosome as a “regulatory Hub” [200] in the cell may clarify how mutations in 13 different *CLN* genes may impair lysosomal function. The endo-lysosomal pathway requires coordinated expression and action of various components that regulate the action(s) of the acid hydrolases, lysosomal acidification machinery, and the lysosomal membrane proteins. Recently, it has been reported that a motif (10 base pair sequence) near the transcription initiation site of many genes, designated as coordinated lysosomal expression and regulation (CLEAR), controls the expression of the genes that encode many lysosomal proteins [12]. The transcription factor EB (TFEB), upon translocation to the nucleus, binds to the CLEAR element and promotes the expression of many genes encoding lysosomal proteins [53]. Indeed, the CLEAR element is found in many genes encoding both non-lysosomal and lysosomal proteins including LAMP1, NPC1 and NPC2, β-galactosidase, CTSD, *CLN3* and *CLN5* [12]. However, TFs other than TFEB may also regulate the transcription of proteins such as progranulin, which is associated with neurodegenerative disorders like FTLD and neurodegenerative LSDs like *CLN11* disease [180]. Notably, in the LSDs, the TFEB translocates from the cytoplasm to the nucleus activating its target genes. However, the phosphorylation of TFEB by mTORC1 prevents its translocation from the cytoplasm to the nucleus. These events attest to the fact that a genetic program regulates lysosomal biogenesis and function [201, 202]. It is noteworthy that the activation of mTORC1 impairs autophagy [199] and dysregulation of lysosomal acidification impairs autophagy in common neurodegenerative diseases like Alzheimer’s [44, 203]. Moreover, mTORC1 activation has been reported in JNCL [84] and recently, it has been shown that TFEB activation by mTORC1-independent pathway can be stimulated by suppression of Akt by trehalose, which appears to be neuroprotective and lifespan expanding in a mouse model of *CLN3*-disease [106]. In virtually all LSDs including most of the NCLs lysosomal acidification is dysregulated [25, 26, 67]. Since dysregulation of autophagy has been suggested as a common mechanism underlying pathogenesis of the LSDs including the NCLs [16], suppression of mTORC1 activation is being considered as a therapeutic strategy for these diseases.

Within less than a decade, the discovery that the lysosome responds to environmental cues by sensing the nutritional status of the cell and regulating both cellular clearance and energy production has opened a new era in lysosomal research. It has been reported that activation of mTORC1 localized on the lysosomal membrane regulates cellular growth and homeostasis [204]. In this regulatory system, the nutrients in lysosomal lumen promote TFEB-phosphorylation catalyzed by mTORC1, which inhibits the activity of TFEB. Notably, inhibition of mTORC1 by its inhibitors like rapamycin, activates TFEB by its translocation to the nucleus. Similar results were obtained when the cells were starved to deplete nutrients like amino acids in the lysosomal lumen. Interestingly, the Rag GTPases in coordination with the v-ATPase-Ragulator complex sense lysosomal amino acids activating mTORC1. Thus, the lysosome appears to sense its luminal content to regulate its own biogenesis by an “inside-out” signaling mechanism, which requires both TFEB and mTOR [54, 205]. Notably, S6K1 is phosphorylated by mTORC1 and in the brain of *Cln1*^*-/-*^ mice elevated p-S6K1 levels have been reported [206] suggesting dysregulation of mTORC1, which may impair autophagy. The mTORC1 is a multiprotein complex, which together with the Rag GTPases, Ragulator, and the v-ATPase, forms an amino acid-sensing machinery on lysosomal surface, which at multiple levels affects the decision between cell growth and catabolism [204]. Moreover, diminished mTORC1-dependent JNK activation in *Drosophila* has been shown to cause neurodevelopmental defect. An elegant review on the roles of mTOR activation in several neurodegenerative diseases has recently been published [205]. Although the mTORC1 signaling in NCL diseases has not been studied extensively, it has recently been reported that mutations in the *CLN7* gene cause depletion of soluble lysosomal proteins, which impair mTOR reactivation [136]. It is likely that mTOR signaling abnormalities may underlie pathogenesis of other NCLs besides *CLN7*-disease.

**Fig. 3:**
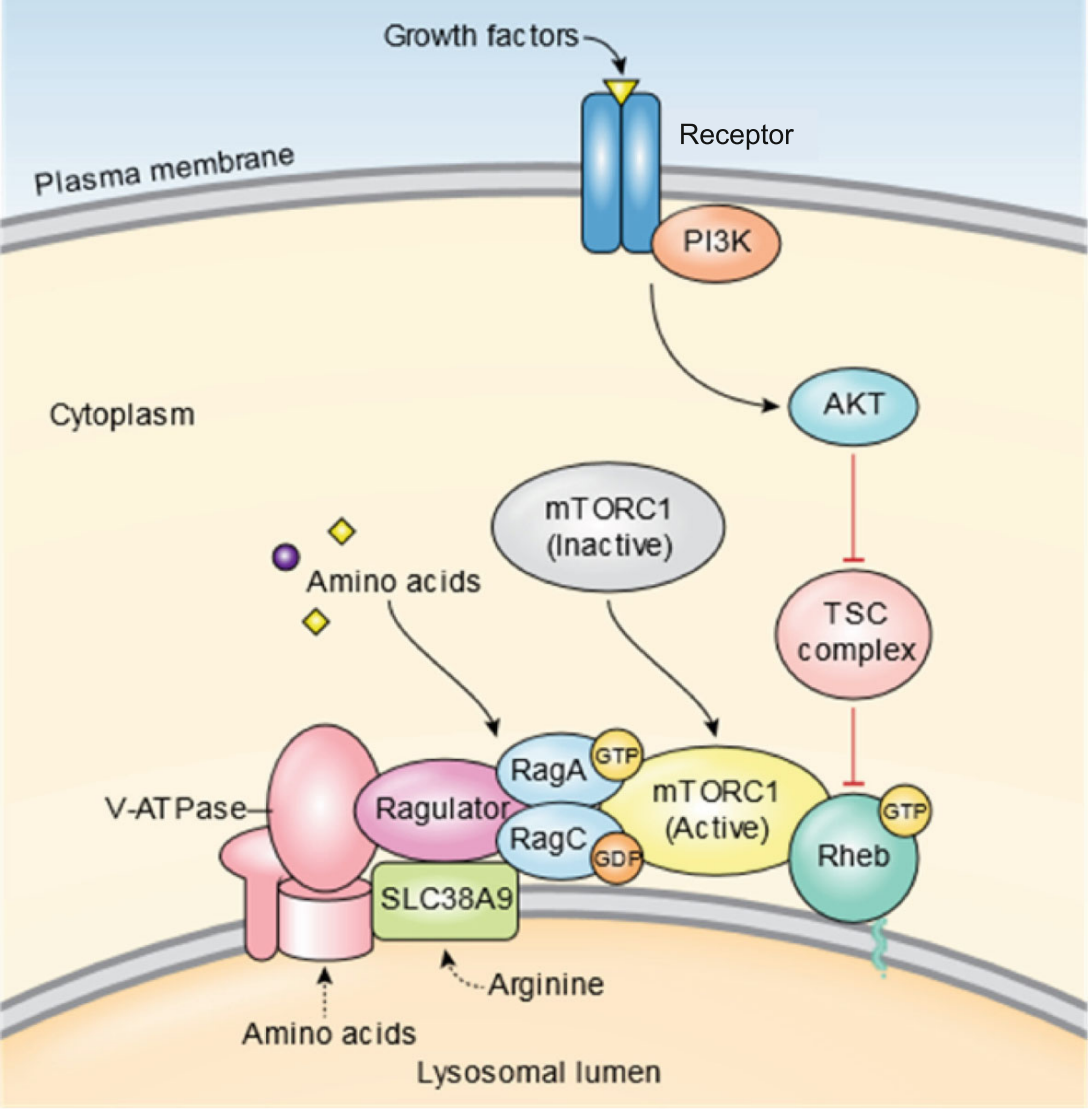
Lysosome as the nutrient sensor and signaling hub of the cell. Emerging evidence indicates that the lysosome in addition to performing its digestive function also acts as a signaling hub for regulating cellular metabolism. In growing cells, signals from amino acids (e.g. arginine, leucine etc), within the lysosomal lumen are integrated upstream of the Rag and Rheb GTPases to promote recruitment of mTORC1 on lysosomal membrane leading to its activation. Signals from other factors such as oxygen and growth factors are also integrated in this fashion by the AKT-TSC pathway. Upon activation, AKT relieves the TSC complex from inhibiting Rheb. The v-ATPase, Ragulator, Rag GTPases and SLC38A9 also participate in the complex process of mTORC1-translocation to the lysosomal membrane where it is activated. Disruption of one or more of these signaling inputs may impair mTORC1 signaling and its recruitment to the lysosomal membrane suppressing its kinase activity. It should be noted that in a nutrient replete state the mTORC1 activation stimulates cell proliferation (anabolic effect), whereas in nutrient depleted state mTORC1 is inactive allowing autophagic pathway to be active (catabolic effect). Most notably, inhibition of mTORC1 by rapamycin and its analogs has been reported to ameliorate pathology and increase lifespan. *Abbreviations used*: mTORC1, mechanistic target of rapamycin complex 1; AKT, Protein kinase B; TSC, Tuberous sclerosis complex; IGFR, Insulin-like growth factor receptor

Recently, it has been uncovered that in Ppt1-deficient *Cln1*^*-/-*^ mice, the misrouting of V0a1 subunit of the lysosomal v-ATPase dysregulates lysosomal acidification [68]. Since v-ATPase on the lysosomal membrane is one of the components of the nutrient sensing machinery which regulates mTORC1 signaling [Fig. 3], it is possible that mTORC1 signaling is defective in all NCLs including the *CLN1*-disease. This is one area of investigation that may provide insight into the pathogenesis of INCL. Interestingly, dysregulated lysosomal pH, which is also reported in many LSDs, impairs mitochondrial function and shortens lifespan in yeast [207]. Cumulatively, these results demonstrate the importance of both cytosolic and lysosomal pH in cellular homeostasis and lifespan.

### Current and emerging therapeutic strategies

Unlike those LSDs, in which the pathologic manifestations occur predominantly in the visceral organs, NCLs pose a significant therapeutic challenge. This is primarily because these diseases manifest mainly in the central nervous system, which is relatively inaccessible due to the blood-brain barrier. Moreover, in most of the NCL forms, the disease onset occurs in early childhood while in others the disease progression is very rapid; for a treatment to be effective, it needs to be initiated as early as possible to prevent significant neuron loss. Because the NCLs are rare diseases, most physicians are unfamiliar with their clinical manifestations. Thus, by the time a genetic diagnosis is firmly established, significant neuron loss has already occurred. Despite these obstacles, progress towards therapeutic development continues to be made.

Among the therapeutic strategies, enzyme replacement therapy (ERT) as well as gene therapy for the *CLN*s are making steady progress. However, small molecules that are non-toxic, cross the blood-brain-barrier and mimic the function of the mutant gene may be a valuable addition to the other therapeutic strategies. Recently, attempts are being made to identify and characterize thioesterases-mimetic small molecules for the treatment of INCL. Since nucleophilic attack cleaves the thioester linkage in S-palmitoylated proteins (constituents of ceroid), it was reasoned that nucleophilic small molecules, such as cysteamine and N-acetylcysteine, alone or in combination, may have therapeutic potential for INCL. These compounds have been tested first *in vitro* using cultured cells from INCL patients [208]. Based on the results of *in vitro* studies, a combination of these compounds has been tested in a clinical trial. While the results showed some modest beneficial effects, the patients eventually succumb to the disease [209]. Further efforts to find more potent thioesterases-mimetic small molecules resulted in the identification of a non-toxic small molecule, *N*-*tert*-(Butyl) hydroxylamine (NtBuHA), which is undergoing pre-clinical testing in *Cln1*^*-/-*^ mice. Treatment of *Cln1*^*-/-*^ mice with oral NtBuHA showed its neuroprotective and lifespan extending effects in these animals [210]. Pre-clinical studies on this small molecule are currently ongoing.

Nonsense mutations in the *CLN1* gene can cause INCL and the availability of nonsense- suppressors prompted the testing of at least one such compound, PTC124 [211]. Previously, PTC124 has been tested *in vitro* using cultured fibroblasts and lymphoblasts from INCL patients to override the premature stop codons resulting from nonsense mutations. However, this treatment yielded only a very modest increase in Ppt1 enzyme activity [212]. This study was followed by the generation of two *Cln1*-knock-in (KI) mouse models carrying a lethal nonsense mutation in the *Cln1* gene commonly found in INCL patients in the US [213, 214]. In one study [213], PTC124 was tested *in vivo* using Ppt1-KI mice, which yielded promising results.

Several ongoing preclinical studies demonstrating the efficacy of Ppt1 enzyme replacement therapy [215–217] as well gene therapy [218, 219] continue to pave the way for clinical trials in INCL patients. Recently, it has been reported that a commonly used GAP junction inhibitor, carbenoxolone (CBX), a compound that has been proposed to modify lipid microdomains and corrects defective membrane fluidity in *Cln3*-deficient endothelial cells, which ameliorates defects in endocytosis, caveolin-1 distribution at the plasma membrane, and Cdc42 activity [220]. Remarkably, treatment of the *Cln3*-deficient mice with CBX improved the status of blood-brain-barrier and reduced autofluorescence. Since CBX has been used in humans it has been suggested that CBX and related compounds may have therapeutic potential for patients with *CLN3*-disease [220]. The current and emerging therapeutic approaches are presented in several excellent articles [221–227]. More specifically, the current therapeutic approaches, which have reached pre-clinical or clinical trials, include *CLN1, CLN2, CLN3* and *CLN6* diseases (Table 3). Both small and large animal models of various forms of NCLs are being developed. These animal models are likely to be very useful for the preclinical evaluation of novel therapeutic strategies. In this regard, a recently published comprehensive review provides an extensive list of small and large animal models of various NCL forms [228]. Detailed information on various clinical trials can be found in http://clinicaltrials.gov.

**Table 3:** A partial list of completed or ongoing NCL clinical trials^a^

NCL-related proteins	Natural History/ Treatment	National Clinical Trial Number	Study Location	Status	Phase
*CLN1*	Single Group Assignment, Procedure: Surgery to implant human CNS stem cells, single dose Drug: Immunosuppression for 12 months post transplant	NCT00337636	Oregon Health and Science University StemCells, Inc.	Completed	Phase 1
	Single Group Assignment, Interventional, Small Molecule, Cystagon and N-acetylcysteine	NCT00028262	NICHD^d^/NIH	Completed	Phase 4
*CLN2*	Single Group Assignment, Biological, ERT (BMN-190 [recombinant human tripeptidyl peptidase-a (rhTPP1/cerliponase alfa)]), 30-300 mg ICV infusion administered every other week for at least 48 weeks	NCT01907087	NCH^b^ University Hamburg-Eppendorf Guy’s St & Thomas NHS Foundation Trust Hospital for NHS Foundation Trust BioMarin Pharmaceutical	Completed	Phase 1/2
	Parallel Assignment, Biological, AAVrh.10CUhCLN2 (either 9.0 × 10^11 or 2.85 × 10^11 genome copies)	NCT01414985	WCMC^c^	Active, not recruiting	Phase 1&2
	Parallel Assignment, Genetic: AAVrh.10CUhCLN2 (either 9.0 × 10^11 or 2.85 × 10^11 genome copies)	NCT01161576	WCMC	Active, not recruiting	Phase 1
	Parallel Assignment, Genetic: AAV2CUhCLN2 (3 × 10^12 particle units)	NCT00151216	WCMC	Active, not recruiting	Phase 1
	Observational, Case-Only	NCT01035424	WCMC	Active, not recruiting	N/A
	Single Group Assignment, Biological: BMN-190, 300 mg ICV infusion administered every other week for up to 240 weeks Device: Intraventricular access device, surgical implantation of an MRI compatible ICV access device in the lateral ventricle of the right hemisphere	NCT02485899	Columbus, Ohio, United States Hamburg, Germany Rome, Italy London, United Kingdom BioMarin Pharmaceutical	Active, not recruiting	Phase 1/2
	Single Group Assignment, Biological: BMN-190 & recombinant human tripeptidyl peptidase-1 (rhTPP1), an age-appropriate dose of BMN 190 administered via intracerebroventricular (ICV) infusion every other week (qow) for a duration of 144 weeks Device: Intraventricular access device, surgical implantation of an MRI compatible ICV access device in the lateral ventricle of the right hemisphere	NCT02678689	Columbus, Ohio, United States Hamburg, Germany Rome, Italy London, United Kingdom BioMarin Pharmaceutical	Enrolling by invitation	Phase 2
	Observational, Natural History, Primary Outcome: correlation analysis between genotype (genetic constitution) and baseline [time frame: 18 months]	NCT00151268	WCMC	Completed	N/A
	Single Group Assignment, Procedure: Surgery to implant human CNS stem cells (HuCNS-SC) Drug: Medication to suppress the immune system for 12 months post transplant	NCT00337636	Oregon Health and Science University StemCells, Inc.	Completed	Phase 1
*CLN3*	Crossover Assignment, Drug: Small Molecule (Mycophenolate mofetil)	NCT01399047	University of Rochester	Completed	Phase 2
	Natural History, Cohort	NCT03307304	NICHD/ NIH	Recruiting	
*CLN6*	Observational/Natural History, Primary Outcome: disease progression [time frame: 3 years]	NCT03285425	NCH	Recruiting	N/A
	Single Group Assignment, *Gene* Therapy, Drug:scAVV9.CB.CLN6 administered by intrathecal injection	NCT02725580	NCH	Recruiting	Phase 1/2
General Batten Disease	Observational [Patient Registry], Cohort, Primary Outcome measures: Refinement and validation of UBDRS, The natural history of Batten Disease [both time frames:10 years]	NCT01873924	University of Rochester	Recruiting	N/A
	Observational, Cross-Sectional Primary Outcome measure: sleep disturbance Secondary Outcome measure: epilepsy onset, Blindness [both time frames: 1 year]	NCT01966757	NCH	Completed	N/A

^a^For a complete list of clinical trials go to: https://Clinicaltrials.gov

^b^NCH, Nationwide Children’s Hospital

^c^WCMC,Weill College of Medicine, Cornell University

^d^NICHD, National Institute of Child Health and Human Development, NIH, National Institutes of Health

### Outlook

The lysosome, with its uniquely acidic pH and acid hydrolases, is the terminal organelle shared by both endocytic and autophagic pathways of degradation. Since the discovery of the lysosome more than six decades ago, tremendous progress has been made towards identifying the mutated genes underlying LSDs. However, the mechanism(s) by which these mutations impair lysosomal function, causing pathogenesis of the neurodegenerative LSDs, remains poorly understood. Unravelling the complexity and molecular mechanisms that underlie the endo-lysosomal system and delineating the nature of the pathophysiology of neurodegenerative disorders in general remain a formidable challenge. Currently a major objective in the treatment of the LSDs and other common proteinopathic neurodegenerative disorders has been the substrate reduction and clearing of the lysosomes. Although this approach has been moderately successful in treating LSDs that involve the visceral organs, it shows limited benefits at best for the neurodegenerative LSDs like the NCLs. While we continue to learn more about the biological functions of the mutant genes, as related to the pathophysiology of these diseases, the development of mechanism-based treatment may be possible. The development of new vectors for gene therapy is another avenue that may be successful in the foreseeable future. The emerging new roles of the lysosome as they relate to the LSDs and common neurodegenerative diseases may further advance our understanding of the pathogenic mechanisms underlying these diseases and facilitate the development of novel therapeutic strategies. Correlation of basic and clinical data from different therapeutic trials may also contribute to our understanding of these diseases and further identify novel therapeutic targets. It is hoped that in the ensuing years, we will be able to address the following basic questions: (i) what are the critical elements that regulate the endo-lysosomal transport system and what makes this system dysfunctional in neurodegenerative LSDs; (ii) what roles do the lysosomal membrane proteins and their post-translational modifications play in regulating nutrient sensing and mTOR-signaling and how are they dysregulated in neurodegenerative LSDs; (iii) what are the critical elements that regulate membrane fusion between endosome-lysosome and autophagosome-lysosome and (iv) what mechanisms underlie dysregulation of lysosomal acidification in virtually all LSDs as well as in common neurodegenerative diseases. It has recently been proposed that since lysosomal acidification is dysregulated in most LSDs, reacidification of the lysosome may be one of the therapeutic approaches to be considered. Finally, we must aspire to understand what therapeutic interventions can counteract the above-mentioned dysfunctions and what interventions may have a positive impact to ameliorate these defects. Our efforts to answer the above questions may enable us to develop effective therapeutics for the neurodegenerative LSDs, which mostly affect children. We anticipate that advances in our understanding of the disease mechanism(s) coupled with the improved methods of restoring normal lysosomal function by small molecules that cross the blood-brain barrier, the development of novel strategies to deliver the missing gene product, and the generation of vectors to deliver gene therapy to the brain may lead to effective treatments for these devastating diseases.

## Conclusion

In summary, endolysosomal and autophagic dysfunction underlie most of the LSDs and neurodegeneration is a devastating manifestation in most of these diseases. Neuronal ceroid lipofuscinoses are the most common neurodegenerative LSDs that mostly affect children. Although the mutant genes underlying each of the 13 NCL forms have been identified and characterized, the physiological functions of the gene products remain poorly understood. Consequently, the pathogenic mechanism(s) of the NCLs remain elusive despite intense investigations. While major advances towards understanding the pathophysiology of the NCLs have been achieved more research is needed to arrive at the finish line. Despite the lack of mechanistic understanding of these diseases, progress is being made towards the development of effective therapies. In this regard, animal models are a very useful tool. Although replacement of the missing gene product and gene therapy approaches have made significant progress, and some are in clinical or pre-clinical trials, efforts to develop mechanism-based therapeutics should continue. Another area of research that needs more attention is the search for biomarkers for each of the NCL forms. The emerging new roles of the lysosome is an area of research that promises to yield new information on all LSDs including the NCLs.

## Abbreviations

CLEAR: Coordinated lysosomal expression and regulation
CLN: Ceroid lipofuscinosis neuronal
CSPα: Cysteine string protein alpha
CTSD: Cathepsin D
ER: Endoplasmic reticulum
ESCRT: Endosomal sorting complex required for transport
GAP-43: Growth associated protein 43
INCL: Infantile neuronal ceroid lipofuscinosis
JNCL: Juvenile NCL
KCTD7: Potassium channel tetramerization domain-containing protein 7
LINCL: Late infantile neuronal ceroid lipofuscinosis
LSDs: Lysosomal storage diseases
mTORC1: Mechanistic (mammalian) target of rapamycin complex 1
NCLs: Neuronal ceroid lipofuscinoses
NPC1: Niemann pick C1
PPT1: Palmitoyl-protein thioesterases-1
SCMAS: Subunit c of mitochondrial ATP synthase
SNAP25: 25-kDa Synaptosomal protein
SNARE: Soluble N-ethylmaleimide-sensitive attachment receptor
SV: Synaptic vesicle
TF: Transcription factor
TFEB: Transcription factor EB;TPP1, Tripeptidyl peptidase 1
UPR: Unfolded protein response
VAMP2: Vesicle-associated membrane protein 2
v-ATPase: Vacuolar adenosine triphosphatase
